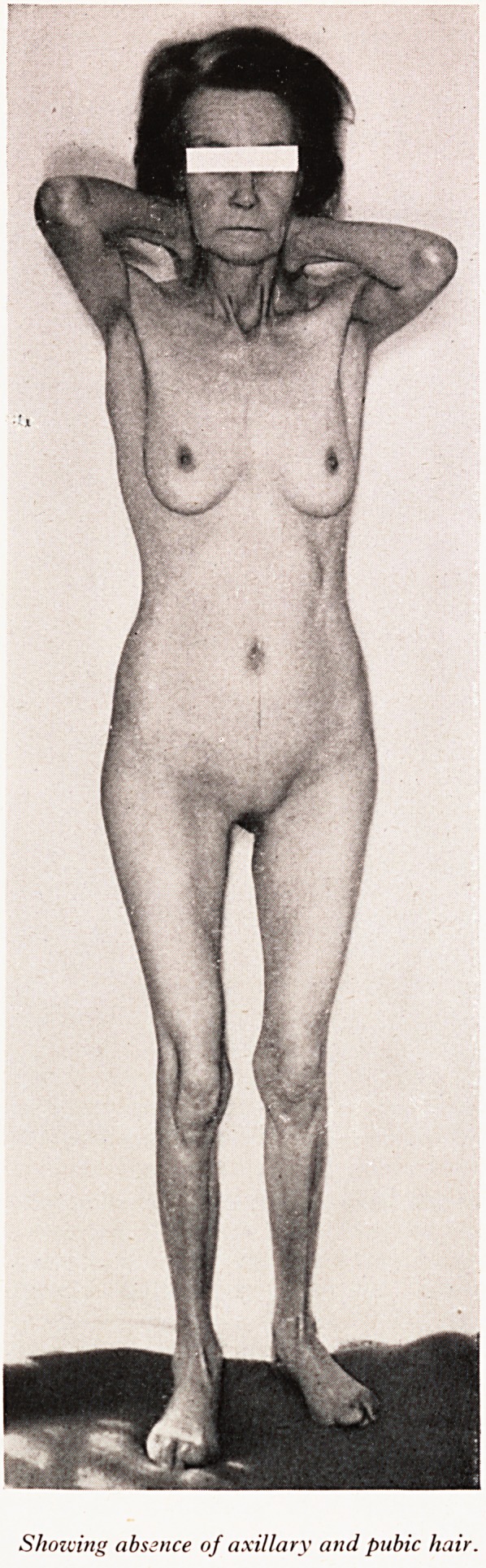# Post-Partum Necrosis of the Pituitary

**Published:** 1960-01

**Authors:** T. F. Hewer


### POST-PARTUM NECROSIS OF THE PITUITARY

*A Clinical Pathological Conference of the University of Bristol Medical School on 5th May, 1959, P.M.* 6038

CHAIRMAN: PROFESSOR T. F. HEWER

*Dr. J. E. Cates:* In 1954, at the age of 58, this woman was admitted into the United Bristol Hospital under the care of Mr. Pocock as an emergency. She came in with bellyache, and her doctor said in a letter that he thought she might have acute intestinal obstruction. For the last few months she had been short of breath on exertion and had been tired and the house surgeon noted that there were some physical signs in the chest, including decreased movement on one side, and there was difference in breath sounds. A chest X-ray showed that the patient had a very large cavity which is considered to be consistent with chronic pulmonary tuberculosis. (See Plate I.)

When I saw her, she presented an astonishing picture: a pale wizened apathetic woman looking a lot older than her years, with no hair in her axillae and virtually none on her pubis; the photograph (Plate II) was taken a little later on, when she was feeling better. She had married when 31 and at the age of 37 she had her first and only baby, a boy. She had a very difficult time with severe haemorrhage afterwards and shortly after that she alleged (wrongly) that there had been so much trouble that her uterus was removed. In fact she did have a uterus, as you will see from the post-mortem notes, but she did not have any more children or periods. She hated the cold and was apathetic and miserable and did not take much interest in what was going on about her. These facts all pointed to her having what we call Sheehan's syndrome (or post-partum pituitary necrosis). She had however escaped three fates which may befall people with anterior pituitary necrosis. She had not been submitted to anaesthetic and been killed, her condition had not been mistaken for pernicious anaemia and treated, nor had it been diagnosed as myxoedema and treated with thyroid.

In spite of her feeble state she did not seem to have *active* tuberculosis, she had no cough, no sputum, she was not coughing up blood and there was no history to suggest active tuberculosis in the past. There was no history of contact. On clinical examination she was not running a fever. Although scraggy, she had not lost weight; she had not been sweating. There was no clubbing and I could not get any sputum out of her at the bedside. The physical signs were in keeping with the X-ray; the movement was down on this side; the trachea was pulled over. Her vocal fremitus was increased and percussion note is noted to have been decreased. There was bronchial breathing over the area corresponding to the cavity shown on the X-ray; there was no post-tussic suction. B.P. was 150/80. All the sputum was negative for tubercle bacilli, and six stomach washings were likewise negative, so I asked Dr. Pearson to see the patient and he thought "she has a very chronic fibroid disease of the upper zone, definitely tubercle—quite likely burnt out. She also had scanty rales at the left base where X-ray also shows some fibrosis". He suggested tomography for further views of the cavity. The tuberculosis therefore did not seem to be active and certainly not infectious so we turned our attention to the endocrine disturbances. Incidentally during the remaining four-and-a-half years of her life her chest was X-rayed every six months and the appearances remained unchanged.

So to return to the picture of panhypopituitarism: we found that she had gross disturbance of electrolytes which is unusual in a pituitary disorder of this nature. Her serum sodium was 113 Meq./L, a very low figure, and her urine sodium was high, 118 at Meq./L, which findings together are very good evidence of suprarenal deficiency. There are many causes of a low sodium but if you find that the kidney is squandering

PLATE I

*X-Ray of chest showing cavitation and fibrosis at the right apex.*

PLATE II

*Showing absence of axillary and pubic hair.*

sodium when the body is short of it you suspect that there is poor function of the suprarenal cortex. The urinary cortico-steroid output was estimated four times and was under 1 mg. for 24 hours. Her thyroid function was decreased; her B.M.R. was 33 per cent. Her E.C.G. was abnormal with *T* waves flat in leads 2 and 3, and low voltages, both of these suggesting that she was hypothyroid.

Another possibility was that if she had tubercle she could have Addison's disease; but X-ray showed no calcification in her suprarenals and the ACTH test for eosinophils, for what it is worth, showed a profound drop. At that time we were not relying on the effect of ACTH on the steroids in urine (we were still studying our methods); I would do this steroid test nowadays for it is the best way to determine whether the suprarenal failure is primary or secondary.

There was also a possibility that she might have a tumour in her pituitary fossa but X-ray showed no evidence of this, neither was there any "neighbourhood" sign or symptom—she had no alteration of her visual fields and no symptoms of headache and so on. So we concluded that after her large post-partum haemorrhage she suffered necrosis of most of her anterior pituitary gland.

As her serum sodium was low she was given 1 g. of sodium chloride t.d.s., together with cortisone acetate with a modest dose of 25 mgm. twice daily; she did not respond appreciably so the dose was increased to 25 mgm. three times a day and then suddenly the picture changed completely: from being apathetic and docile, dull, pale, she became maniacal. She picked up jugs from which she was fed by nurses and smashed them against the wall, and really alarmed everybody; she also became persecuted—a dramatic change with just a few grammes of cortisone. So Dr. Barbour was called in; he was most interested. Cortisone was stopped and she was sent out on extra salt by mouth only, and we watched her in out-patients' department. It was soon quite clear, however, that she needed steroid treatment because within a few weeks she became even more depressed and apathetic again and developed for the first time what is a really dangerous complication with hypopituitarism, namely diarrhoea. The danger is that diarrhoea is a drain on the salt and water reserves of the body and imposes a strain on the ineffectual suprarenals. When other patients with panhypopituitarism have gone into a coma there has often been preceding diarrhoea. She was re-admitted and started on 6·25 mg. of cortisone b.d., and because she was post menopausal and skinny we gave her melthyl testosterone, 10 mgm. a day, and thirdly, because she was still squandering her salt I gave her a D.O.C.A. implantation. To cut a long story short, each time she was seen in the endocrine clinic after that she was remarkably well, cheerful, active and physically very much better. Her electrolytes were almost satisfactory, and apart from an episode in which she was discovered to have broken ten ribs, presumably from cough fractures, in 1956, her course was eneventful until just before she died. On the day of her death, doctor rang and said "Mrs. L. with panhypopituitarism has got a chest infection and is very poorly, will you admit her?" I said "send her in an ambulance". She was sent straight in, but she died in the corridor between the surgical and medical blocks as she was being admitted.

All patients with suprarenal deficiency from any cause are taught that they need a much larger dose of cortisone during a severe infection. This lady seems to have died within a few hours of becoming ill.

*Professor Hewer:* Has anyone any questions for Dr. Cates at this stage?

*Question:* Was she treated with thyroid extract and what is your view of this treatment?

*Dr. Cates:* No. She did not need it clinically.

*Question:* Did you put her on streptomycin?

*Dr. Cates:* No. There was no evidence that she had active tubercle.

*Question:* Is cortisone therapy likely to re-activate the tubercle?

*Dr. Cates:* If you give cortisone in what is dramatically called "poisonous doses" to suppress say, rheumatoid arthritis, patients are more likely to develop infection, but where you are just replacing their day to day needs, then they are in no more danger of this than we are.

*Dr. Allwood:* What was her E.S.R.?

*Dr. Cates:* In 1954 she had a Hb of 70 per cent; E.S.R. 38.

*Question:* Is there any advantage in giving A.C.T.H. to this patient.

*Dr. Cates:* No. A.C.T.H. is a protein and proteins stimulate the production of antibodies so A.C.T.H. may lose its effect and moreover, prolonged A.C.T.H. treatment is more expensive. Lastly if you give replacement treatment it is not an injection and patients prefer it.

*Question:* Was not her blood pressure unexpectedly high throughout her first hospital admission?

*Dr. Cates:* Yes, but it went up very much higher when she was treated; it went up to 220/100 when she was "normal" later on.

*Question:* Did she then have hypertension?

*Dr. Cates:* I think she was a hypertensive woman. Her B.P. fell to 150/80 when she was ill.

*Dr. Betteridge:* We heard what her general appearance was like before she commenced treatment. As a result of treatment she did have some quite reasonable amount of pubic hair; she had some axillary hair, her eyebrows were quite complete and the hair of her head was not at all sparse. However, she was very thin. She was 5 ft. tall but she weighed just 5 stones, which is well below her expected weight. Another point of interest was that the pigmented areas, which are normally said to be de-pigmented in cases of panhypopituitarism, were in fact now normally pigmented.

The pituitary gland was small with obvious loss of anterior lobe substance. The posterior lobe appeared normal. Microscopically there was only a small fraction of functioning anterior lobe present, lying just in front of the pars intermedia. There were no acidophils present and few basophils, many of which were hyaline. Chromophobe cells were present and appeared normal.

The adrenal glands were small, as might be expected, their total weight being 4·5 g., compared with an expected weight of over 7 g. On section the cortex was seen to be reduced to between 0·4 and 0·8 mm. in thickness and contained a moderate amount of lipid. Microscopically there was thickening of the capsule with some fibrosis in the zona glomerulosa, which alone of the zones of the cortex could be recognized with any certainty since there was general disorganization of the cortex. In some areas the zona glomerulosa could be recognized adjacent to the medulla which was itself normal.

The thyroid gland weighed only 7 g., just about a quarter of the normal expected weight, and was flattened and fibrous in appearance. Microscopically there was considerable diffuse fibrosis present without round cell infiltration. The acini were lined by flattened cuboidal epithelium and contained colloid.

The heart weighed only 180 g. and was reduced in size but otherwise was externally normal. There was very little atheroma present in the main vessels and the aorta itself was surprisingly elastic. Microscopically the heart muscle fibres were small and contained lipochrome pigment and a little fat.

The liver was greatly reduced in size, weighing only 695 g. The cells appeared reduced in size microscopically.

The kidneys (weight 105 and 100 g.) were normal with no evidence of arteriosclerosis. The uterus was present and atrophic, as were the ovaries.

The other main findings of interest in this case were in the lungs. The left pleural cavity was obliterated by hard fibrous adhesions in its upper part; in the lower part there were recent soft fibrinous adhesions with loculi of clear serous fluid. The upper lobe of the left lung showed marked old apical fibrosis, and the so-called "sugar icing" appearance of thickened hyaline visceral pleura extending into the interlobular fissure. The lower lobe showed scattered areas of old fibrosis and recent bronchopneumonia. The right pleural cavity was obliterated by firm adhesions. The right lung showed a similar thickening of the visceral pleura of the upper lobe which was itself entirely replaced by old thick-walled cavities which contained thick creamy flocculent material. Two of the cavities were shown to communicate with bronchi which showed a moderate degree of bronchiectasis. The little lung tissue present was fibrous. The middle lobe showed a confluent pneumonia and the lower lobe had areas of old fibrosis and recent bronchopneumonia similar to that seen in the left lower lobe. The microscopical appearances of the old lesions and the cavities were of non-specific fibrosis. There was no unequivocal evidence of tuberculosis. No tubercle bacilli were present. Culture of the lungs revealed a good growth of pneumococci.

In short, this was a woman who survived post-partum necrosis of the anterior lobe of her pituitary to show the features of panhypopituitarism with secondary deficiency of other endocrine glands. It is interesting to note the absence of cardiac hypertrophy and renal lesions in the presence of a sustained hypertension. Her chronic chest condition had all the feature of a tuberculous process which had "burnt out", and she died finally of an overwhelming pneumococcal bronchopneumonia.

*Professor Hewer:* Dr. Cates, what is your interpretation of the blood pressure reading. I believe the highest diastolic blood pressure recorded was 105, would you be inclined to call this hypertension with a systolic pressure of 180 at one time?

*Dr. Cates:* It was above that figure. I would agree with you that she had hypertension. Some of the other patients with pituitary failure on replacement therapy had had high blood pressure. It may be that we have not yet struck the right balance of hormone replacement.

*Professor Hewer:* I would like to follow up that question by asking whether cortisone was responsible for what rise there was.

*Dr. Cates:* It may well be so. She was a small woman and was otherwise sensitive to normal therapeutic doses.

Her fractured ribs are interesting. Dr. Betteridge did not mention that; but in pituitary necrosis the lack of androgens from the suprarenals and oestrogens from the ovaries in a woman can lead to decalcification, for that reason we gave her small doses of testosterone. Dr. Betteridge, can you tell us whether the bone has been examined?

*Dr. Betteridge:* Yes, sections of a vertebra and of the ribs show definite evidence of osteoporosis.

*Professor Hewer:* It is a pretty classic example, is it not, of pituitary necrosis, following post-partum haemorrhage and the panhypopituitarism which follows.

*Dr. Cates:* Yes. Presumably with just enough anterior pituitary gland remaining, 10 per cent or so, to keep her alive, just enough to prevent complete failure of her suprarenals and thyroid. If the pituitary is completely destroyed patients do not live long.

## Figures and Tables

**Figure f1:**
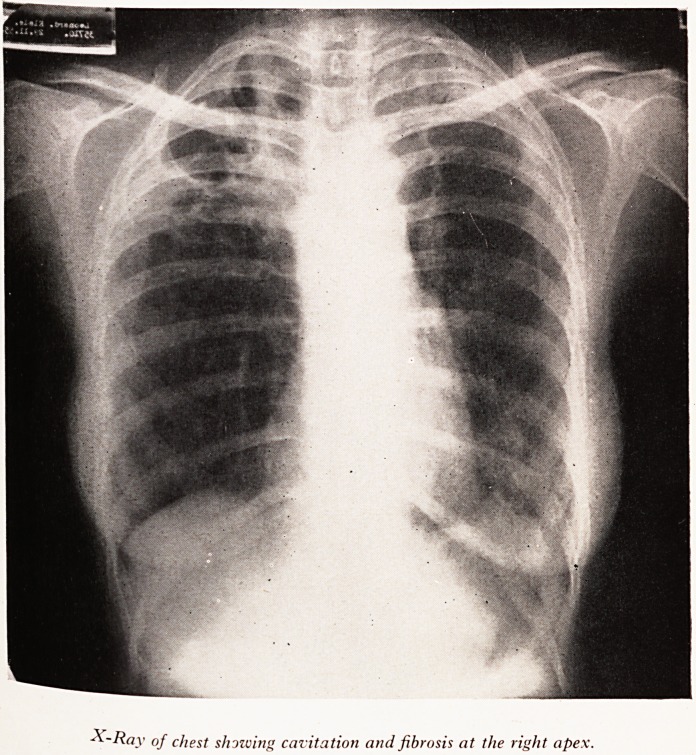


**Figure f2:**